# Exploring the Possibility of Measuring Vertebrae Bone Structure Metrics Using MDCT Images: An Unpaired Image-to-Image Translation Method

**DOI:** 10.3390/bioengineering10060716

**Published:** 2023-06-12

**Authors:** Dan Jin, Han Zheng, Huishu Yuan

**Affiliations:** 1 Department of Radiology, Peking University Third Hospital, Beijing 100191, China; jindan1418@bjmu.edu.cn; 2 School of Traffic and Transportation, Beijing Jiaotong University, Beijing 100044, China; hanzheng@bjtu.edu.cn

**Keywords:** micro-CT-like images, unpaired image-to-image translation, vertebrae, bone structure

## Abstract

Bone structure metrics are vital for the evaluation of vertebral bone strength. However, the gold standard for measuring bone structure metrics, micro-Computed Tomography (micro-CT), cannot be used in vivo, which hinders the early diagnosis of fragility fractures. This paper used an unpaired image-to-image translation method to capture the mapping between clinical multidetector computed tomography (MDCT) and micro-CT images and then generated micro-CT-like images to measure bone structure metrics. MDCT and micro-CT images were scanned from 75 human lumbar spine specimens and formed training and testing sets. The generator in the model focused on learning both the structure and detailed pattern of bone trabeculae and generating micro-CT-like images, and the discriminator determined whether the generated images were micro-CT images or not. Based on similarity metrics (i.e., SSIM and FID) and bone structure metrics (i.e., bone volume fraction, trabecular separation and trabecular thickness), a set of comparisons were performed. The results show that the proposed method can perform better in terms of both similarity metrics and bone structure metrics and the improvement is statistically significant. In particular, we compared the proposed method with the paired image-to-image method and analyzed the pros and cons of the method used.

## 1. Introduction

Bone mineral density (BMD) tests are now internationally recognized as the primary method of diagnosis for vertebral fragility fractures [1,2]. However, even with standardized image quality requirements, diagnostic criteria and operating manuals, the rate of underdiagnosis of fragility fractures remains high [3,4,5,6,7,8,9]. A high rate of underdiagnosis means that patients miss out on the timely treatment of vertebral fractures, which can lead to height loss, kyphosis, chronic back pain and back-related dysfunction and can significantly reduce the chance of survival of patients.

Numerous studies [10,11,12,13] have found that changes in bone structure decrease bone quality and increase the risk of fragility fractures, suggesting that bone structure also plays a key role in bone strength. For example, Taes Y et al. [14] concluded that fractures in adult men are associated with a smaller cortical bone area and reduced cortical thickness, but not with bone density. Wehrli FW et al. [15] studied the bone structure of the distal radius and tibia in postmenopausal women and found that changes in bone structure explained 96% of the change in bone strength, with trabecular volume alone explaining 37–67% of the change in bone strength. Koester et al. found that increased cortical porosity may lead to a 75% reduction in proximal femur bone strength and that cortical porosity increases with age [11]. When the trabecular structure deteriorates, the trabeculae decrease in number, thin or even disappear; gaps widen; and trabeculae transform from plate-like to rod-like; these changes increase separation and decrease connectivity, which ultimately lead to significant changes in structure metrics [10,11,12]. Bone structure includes the macrostructure and microstructure of bone [16]. The macrostructure refers to the geometry and topology of bone, and the microstructure refers to the thickness and spatial distribution of cortical and trabecular bone. For convenience of expression, “bone structure” in this paper refers to the microstructure of bone.

Microcomputed tomography (micro-CT) is the gold standard for measuring bone structure metrics and has a resolution of 10 μm or less. However, it cannot be used to measure bone structure at anatomical sites such as the spine and hip due to the small aperture (<10 cm) and high radiation dose. Multidetector computed tomography (MDCT) can be used routinely for measuring the bone density of human medial bones with calibrated body models, which has a wide range of clinical applications. However, due to the low resolution (approximately 200–500 μm), which is much larger than the average thickness of bone trabeculae, MDCT cannot capture the detailed information of bone trabeculae and therefore cannot support the accurate measurement of bone structure metrics. If the relationship between MDCT and micro-CT images can be obtained using deep learning techniques, it will be possible to generate micro-CT-like images on the basis of MDCT, which in turn enables the measurement of bone structure metrics.

**Figure 1:**
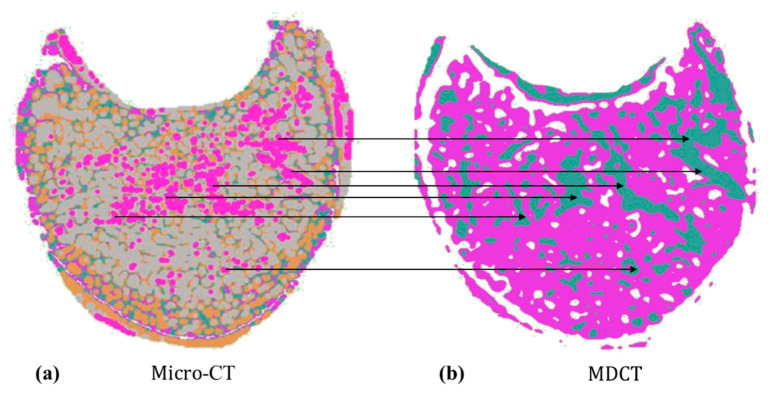
Example of an inherent mapping relationship between micro-CT (**a**) and MDCT (**b**) after the clustering process. Arrows represent the spatial mapping between MDCT and micro-CT images.

The generation of micro-CT-like images from MDCT images themselves has logical self-consistency. Clustering techniques allow us to observe the structural matchings in MDCT and micro-CT images (as shown in Figure 1). The distribution of bone and bone marrow tissues has an obvious spatial mapping. Therefore, it is reasonable to assume that there is also a hidden relationship between low-resolution MDCT and high-resolution micro-CT images in terms of image structure and detail.

Conditional generative adversarial networks (CGANs) [17,18,19,20,21] are currently popular image translation and generation methods. Among these methods, the paired-image-based method has been proven to generate realistic images with sharp details and to have good quantitative performance [22]. Such methods are trained on a paired-image dataset, where an image from the source domain already has a corresponding translated image in the target domain. In the domain of our study, the paired-image-based method requires a large number of paired MDCT and micro-CT images, and finer results can be obtained when a sufficient number of paired samples is obtained. However, this paired dataset requirement imposes a huge practical constraint in the medical field, because micro-CT images can only be obtained from human cadaver specimens. In contrast, the unpaired-image-based method can be trained based on unpaired MDCT and micro-CT images, and the method is less difficult to preprocess than the paired-image-based method.

This paper utilized a method to generate micro-CT-like images from MDCT images using FUNIT [23], a few-shot unpaired-image-based method that enables high-resolution image translation between image domains. This method does not change the clinical scanning technique and measures bone structure metrics that are highly correlated with those of micro-CT images without increasing the cost or radiation dose.

The remainder of the article is organized as follows: in Section 2, we review the history of medical image translations and analyze the need for few-shot unpaired-image-based learning. In Section 3, we systematically present a series of techniques used to measure bone structure metrics. In Section 4, we compare the generation results of the selected method with those of other methods and analyze the properties of unpaired-image-based learning for micro-CT-like image generation.

## 2. Literature Reviews

For measuring bone structure metrics, image translation methods are used to find associations between MDCT and micro-CT images and generate micro-CT-like images. Such methods have been used and explored in the medical field for numerous applications, such as replenishing missing images [24], cross-scan mode conversion [25], image resolution enhancement [26] and creating labeled datasets [27]. Mathematically, the goal of image translation is to transform the input image $x_{A}$ from the original domain A to the target domain B, thus acquiring the detailed features of domain B, while preserving features of the source domain. To achieve this goal, a model $G_{A\to B}$ needs to be trained to generate image $x_{AB}\in B$ given the original domain image $x_{A}\in A$. The generated image cannot be distinguished from the image $x_{B}\in B$ of the target domain. This process can be expressed as follows:

(1) $$x_{AB}\in B:x_{AB}=G_{A\to B}\left(x_{A}\right)$$

In early studies, translation models $G_{A\to B}\left(A\right)$ were implemented via classical image scaling, including four major categories of interpolation methods, frequency domain analysis, instance-based methods and nonlinear learning methods [28,29,30]. The interpolation methods can be further divided into various specific methods, such as nearest neighbor interpolation [31], bilinear interpolation [31] and bicubic interpolation [32,33]. These methods translate images by filling pixels based on the inter-relationship of pixels after expanding the source image to make the image edges and content clear. Frequency domain analysis methods, such as Fourier sharpening and wavelet denoising, Ref. [34] have also gained wider application in the clinical field [35] and have been applied in low-dose X-ray image resolution enhancement. Example-based methods [36] obtain the relationships between regions to achieve image translation. These methods are good at image translation tasks with regular content, such as the resolution improvement of architectural pictures. In addition, nonlinear learning methods, such as dictionary learning [37] and random forest [38], are used in translating medical images, which are based on features selected by experts. However, manually selected features are limited in their ability to represent complex image information in medical image translations. The aforementioned methods are mainly focused on filling in the pixels of the target image (CT or MRI image), which only ensures a clearer image and makes the boundaries between tissues (i.e., edges or contours) clearer and does not extend and fill in the content or structure details [31,39]. Deep learning [40,41] methods can address this problem by automatically learning features.

Deep learning super-resolution methods [42,43,44,45] became popular in medical image translations during 2015 [46]. Convolutional Neural Networks (CNNs) are a dominant class of method [47,48,49,50]. CNNs mimic the way the biological visual cortex works [51] and can be simply understood as the extraction of the boundaries between neighboring pixels by using convolutional kernels. Based on these, CNNs were first used for image translation studies within the same scan pattern. Chen et al. [47] proposed a three-layer CNN model to generate relatively high-quality images from low-dose, low-quality CT images of the human body. Chen et al. [48] used a residual CNN model to achieve low-dose CT image resolution enhancement. These studies provided solutions to effectively reduce the radiation dose of CT scans. In the field of MRI, CNN-based image translations have also been used for image resolution enhancement: Zend et al. [49] used ResNet [52] for the resolution enhancement of brain MRI. Chaudhari et al. [50] used a similar approach to study the resolution enhancement of knee MRI images. These studies provided solutions to effectively reduce the scanning time of MRI and lay the technical foundation for the implementation of image post-processing techniques such as 3T to 7T. In addition, more complex CNN models, such as cascaded CNNs [53], have been explored [54] and applied to more complex medical image mapping tasks. For example, Xiang L et al. [55] investigated the conversion method of T1-weighted images to CT images in cranial MRI via a CNN.

However, CNNs tend to use deeper and higher-dimensional models to obtain a larger perceptual field, which makes the model difficult to train and easy to overfit [56]. At the same time, CNN training aims to minimize the loss function, which tends to focus on minimizing the reconstruction error, and the results may have a high peak signal-to-noise ratio and tend to lose high-frequency details [57,58]. This makes CNN-based methods prone to problems such as blurring and noise on edges and detailed textures, and, in general, only able to handle lower-resolution images. The emergence of Generative Adversarial Networks (GANs) [59] and Conditional Generative Adversarial Networks (CGANs) [60] has provided a new solution for this problem, and these networks have achieved promising results [61,62,63]. These models introduce the concept of adversarial learning based on the powerful feature extraction capability of CNNs and separate the image generation task from the discriminator task to reduce the overall training difficulty.

CGAN-based image translation [18,19,20,59,64] focuses more on the acquisition of internal mapping relationships between different images [19] and the generation of gold-standard-like images, rather than focusing on simple pixel-based filling or sampling, and tends to be better at content connecting and filling [20]. After years of development, the CGAN and its various derivative models have proven suitable for implementation in image translation and have gained widespread attention [30,31,32,33,34]. These methods have been used [22,50,65,66] in medical imaging. For example, Nie et al. [67] used a cascade GAN technique to implement brain and pelvic MRI to generate corresponding CT images and to accomplish the task of 3T MRI to 7T MRI; Hiasa et al. [68] implemented the process of mapping T1-weighted imaging from the pelvis to the distal femur to CT images via CycleGAN. Dar et al. [69] used the pix2pix (a CGAN-derived model) technique [19] to achieve mapping between T1-weighted images and T2-weighted images.

It is worth noting that CGAN-based image mapping methods can be divided into paired-image-based methods and unpaired-image-based methods. Paired-image-based methods [19,22,53,56] aim to train generators and discriminators based on paired-image training sets to achieve “image-to-image” mapping from the source domain to the target domain, while unpaired-image-based methods [20,23,70] aim to train generators and discriminators to achieve “class-to-class” mapping from the source domain to the target domain based on an unpaired (but containing both the domain and target domain images) training set. Because of this, paired-image-based methods require complex collection and preprocessing for images (images from different image domains need to be collected with the same scan pattern as much as possible, and images need to be paired one by one), while unpaired-image-based methods have relatively simple preprocessing steps and do not require pairing.

Generally, paired-image-based methods can obtain results with high similarity to the gold standard if the training dataset is sufficient [56]. However, the image translation studied in this paper requires in vitro data samples, which are generally collected through cadaver specimens for training and testing. It is difficult to collect large-scale data from various aspects, such as policies, regulations and costs. In addition, a large amount of the CT image pairing work itself is costly, which further hinders the scaling up of paired-image-based methods.

Thus, we need a few-shot unpaired-image-based method that can discover the relationships between MDCT and micro-CT images to capture the overall and local multi-resolution features and achieve the accurate generation of vertebral structure and bone trabecular details in a way that supports the measurement of bone metrics. This is still a challenge for imaging methods with large differences and large image sizes such as MDCT and micro-CT.

## 3. Methodology

**Figure 2:**
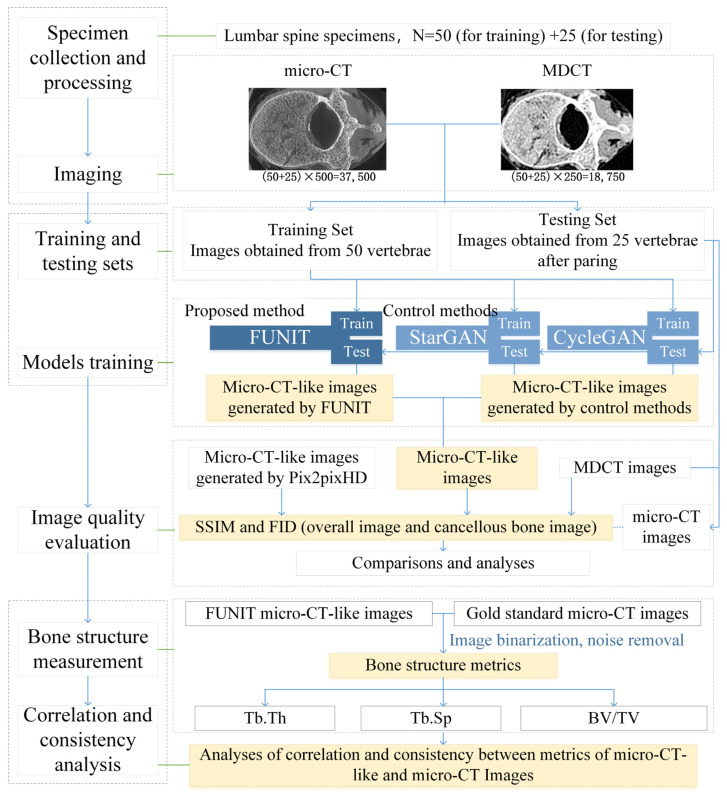
The framework of this study.

Based on the above discussion, a series of techniques related to image translation were designed in this paper based on a few-shot unpaired-image-based method, and FUNIT [23] was applied as the core. To demonstrate the effectiveness of the chosen method, the unpaired-image-based StarGAN [70] and CycleGAN [20] and paired-image-based pix2pixHD [22,56] methods were selected as the control methods. SSIM and FID metrics and vertebral bone structure metrics, including bone volume fraction (BV/TV), trabecular thickness (Tb.Th) and trabecular spacing (Tb.Sp), were measured to demonstrate the feasibility of measuring vertebrae bone structure metrics using MDCT images. The framework of the methodology is shown in Figure 2.

This study was an applied basic research study based on scanned images of human cadaveric lumbar spine specimens. The specimens used were from the Department of Anatomy and Research, Faculty of Medicine, Peking University. All donors signed an agreement related to the donation of human remains and agreed that the remains would be used for clinical medical education and research. The study protocol was approved by the Medical Science Research Ethics Committee of Peking University Third Hospital; the ethics number is IRB00006761-M2021179.

### 3.1. Specimens

In this study, a total of 75 lumbar vertebrae, comprising 15 sets of lumbar spines (L1 to L5), were obtained from 15 formalin-fixed human cadavers (9 males and 6 females; mean age 73 years; age range 62–88 years). These donors had bequeathed their bodies to the local Institute of Anatomy for educational and research purposes, adhering to the relevant institutional and legislative guidelines. Lumbar vertebrae that showed significant compression fractures, bone neoplasms or other substantial bone destruction were excluded from the study. Consequently, all 75 specimens were incorporated into the experiment. The lumbar spine, along with the surrounding muscle, was sectioned into individual segments using a band saw, ensuring the preservation of the pedicle and appendix structures to the greatest extent possible. To minimize trapped gas, the samples were submerged in a phosphate-buffered saline (PBS) solution at 4 °C for a duration of 24 h prior to scanning. The study protocol underwent review and received approval from the local institutional review boards.

### 3.2. Imaging Techniques

The specimens underwent scanning using both micro-CT (Inveon, Siemens, Erlangen, Germany) and MDCT (SOMATOM Definition Flash, Siemens, Erlangen, Germany) imaging techniques. For micro-CT imaging, the parameters were set at 80 kVp/500 mAs, with a field of view on the x−y plane measuring $80\times 80\;mm^{2}$. A standard matrix size of 1536×1536 pixels was employed, along with 1024 slices at an effective pixel size of 52 μm. The exposure time for each of the 360 rotational steps was 1500 ms. In contrast, the MDCT imaging parameters included 120 kVp/250 mAs, a field of view of $100\times 100\;mm^{2}$, a slice thickness of 0.6 mm, a slice spacing of 0.1 mm, a pitch of 0.8 and a standard matrix size of 512×512 pixels. After excluding images with incomplete, upper and lower endplate views, for all lumbar spine specimens, axial images were captured 1.25 cm above and below the center of the vertebral body. Given that the slice spacing for micro-CT was approximately 0.05 mm and the MDCT slice spacing was approximately 0.1 mm, 500 micro-CT images and 250 MDCT images were captured for each vertebra.

### 3.3. Few-Shot Unpaired-Image-Based Translation Model for Generating Micro-CT-like Images

The few-shot unpaired-image-based model, FUNIT [23], learns image mapping relationships from unpaired MDCT and micro-CT images. The model simultaneously learns geometric characteristics, internal structures and the distribution of light and dark regions from MDCT images, as well as the detailed texture of bone structures from micro-CT images. After training, the model can generate high-resolution micro-CT-like images with MDCT images as input.

The model mainly consists of two core modules, namely, (1) a structured detail-filled generator G and (2) a multitask adversarial discriminator D. The generator G can extract micro-structure information and generate gold-standard-like images by filling textures, while the discriminator D can discriminate whether the generated image belongs to the target domain. As an unpaired-image-based learning model, the model is designed to translate among multiple types of images. Mathematically, the generator G takes x and K mapping targets $\left\{y_{1},\cdots ,y_{K}\right\}$ as inputs and outputs generated images $\overset{-}{x}$ with features of K targets.

(2) $$\overset{-}{x}=G\left(x,\left\{y_{1},\cdots ,y_{K}\right\}\right)$$

The low-resolution MDCT is considered to be the input image x. Some high-resolution images such as HR-pQCT [71], micro-CT [72,73,74], etc., can be treated as the mapping targets $\left\{y_{1},\cdots ,y_{K}\right\}$. In this paper, we only consider generating micro-CT-like images, so we set K=1, and the micro-CT image is the only y. Thus, Equation (2) can be written as Equation (3).

(3) $$\overset{-}{x}=G\left(x,y\right)$$

**Figure 3:**
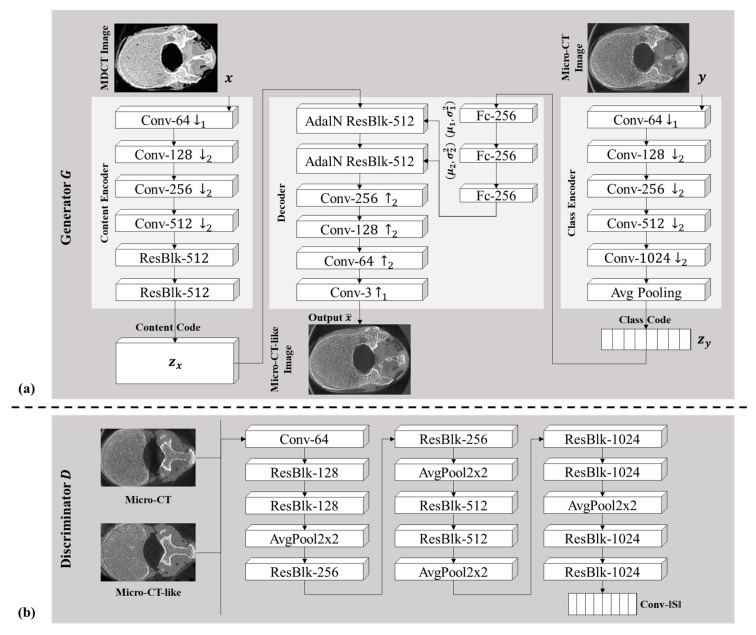
Architecture of (**a**) the generator G and (**b**) the discriminator.

In Equation (3), the generator G is designed to have the ability to generate micro-CT-like images from MDCT. It consists of three sub-networks, namely, the content encoder $E_{x}$, class encoder $E_{y}$ and decoder $F_{x}$, as shown in Figure 3a. 

The content encoder $E_{x}$ is designed to extract texture-independent positional and structural region information, such as the extraction of the vertebral geometry and trabecular layout of the bone. $E_{x}$ consists of two-dimensional convolutional layers and residual blocks [52,75], and each convolutional layer has normalized functions and ReLU nonlinear functions. The feature maps are scaled by a factor of 2 in each spatial dimension using the nearest-neighbor up-sampling method. The input MDCT image is mapped into a spatial feature map $z_{x}$ by a 3-stride-2 down-sampling operation.

The class encoder $E_{y}$ mainly extracts detailed characteristics such as bone trabeculae texture and alignment. It consists of several two-dimensional convolutional layers, which are then averaged along the sampling axis. $E_{y}$ maps the micro-CT images to a class latent code for describing the texture characteristics of bone trabeculae. This process uses a VGG [57] network to map each input class image to a class latent code $z_{y}$. Afterwards, the class latent code is fed to the decoder $F_{x}$ through the AdaIN layer, where $E_{y}$ can control detailed characteristics (e.g., texture) and $E_{x}$ can determine regional characteristics (e.g., the location of regions with different trabecular characteristics). This enables the generation of bone structure details on the basis of reasonable correspondence between MDCT and micro-CT.

The decoder $F_{x}$ takes latent code $z_{y}$ as input and obtains a set of mean and variance $\left(\mu_{i},\sigma_{i}^{2}\right)i=1,2$ through two fully connected networks. These values are then used as affine transformation parameters in the AdaIN residual block, where the $\sigma_{i}^{2}$s are the scaling factors and the $\mu_{i}$s are the biases [76]. For each residual block, the same affine transformation is applied to each spatial location in the feature map. The affine transformation is spatially invariant and therefore can only be used to obtain global appearance information, which controls how the content is potentially encoded for decoding to generate the output image.

According to the above design, the generator G can map the input MDCT image x to the output micro-CT-like image $\overset{-}{x}$ such that $\overset{-}{x}$ looks like an image belonging to the class $c_{y}$ of gold-standard micro-CT images, and $\overset{-}{x}$ and x have structural similarity.

The chosen discriminator D is a patch discriminator [19]. This discriminator applies a Leaky ReLU nonlinear activation function and consists of a convolutional layer and 10 activated residual blocks without normalization [77]. The architecture of the discriminator is shown in Figure 3b. It consists of Conv-64 → ResBlk-128 → ResBlk-128 → AvgPool2x2 → ResBlk-256 → ResBlk-256 → AvgPool2x2 → ResBlk-512 → ResBlk-512 → AvgPool2x2 → ResBlk-1024 → ResBlk-1024 → AvgPool2x2 → ResBlk-1024 → ResBlk-1024 → Conv-$\left\|S\right\|$, where ResBlk-X denotes the residual block of output size X×X [52] and $\left\|S\right\|$ is the number of mapped target image classes, which is two in this study, namely, MDCT and micro-CT images.

### 3.4. Training and Testing

#### 3.4.1. Training Process

The training process of the FUNIT model is a process of solving the minmax optimization problem with the objective function of:

(4) $$\underset{G}{\min}\underset{D}{\max}L_{GAN}\left(D,G\right)+\lambda_{R}L_{R}\left(G\right)+\lambda_{F}L_{F}\left(G\right)$$

where $L_{GAN}$, $L_{R}$ and $L_{F}$ are the GAN loss function, the loss function of the reconstructed input image with the original input domain and the feature matching loss function, respectively. These functions are defined as follows:

(5) $$L_{GAN}\left(D,G\right)=E_{x}\left[\log D^{c_{x}}\left(x\right)\right]+E_{x,\left\{y_{1}\right\}}\left[\log\left(1-D^{c_{y}}\left(G\left(x,\left\{y_{1}\right\}\right)=\overset{-}{x}\right)\right)\right]$$

where $D\left(x\right)$ is a discriminant probability distribution of a discriminator expressing the probability of classifying x as a target gold-standard image, rather than a generated gold-standard-like image, and the superscript indicates the type of target discriminated. That is, $D^{c_{x}}\left(x\right)$ expresses the ability to discriminate the input image as an MDCT image, while $D^{c_{y}}(\overset{-}{x})$ is the ability to discriminate the generated gold-standard-like image as a micro-CT image, and $1-D^{c_{y}}(\overset{-}{x})$ expresses the ability to discriminate the generated gold-standard-like image and not discriminate it as a micro-CT image.

Thus, $L_{GAN}\left(D,G\right)$ expresses the ability of the model to discriminate the input image as an MDCT image and the generated class image as not a micro-CT image. For the discriminator D, the input should be discriminated as an MDCT image and the generated gold-standard-like image should be discriminated as not a micro-CT image as much as possible, so this ability is as large as possible and is taken as max; meanwhile, for the generator G, this ability is as small as possible and is therefore taken as min.

In addition, $L_{R}$ can help train the generator G model for image mapping. Specifically, when using the same MDCT image as the input image and the mapped target image (in this case, K=1), this loss function encourages G to produce an output image that is identical to the input MDCT.

(6) $$L_{R}\left(G\right)=E_{x}\left[{\left\|x-G\left(x,\left\{x\right\}\right)\right\|}_{1}^{1}\right]$$

The $L_{F}$ provides a normalization ability to the training. By removing the last layer of the discriminator D, a feature extractor $D_{f}$ is obtained. Using $D_{f}$ to extract features from the class micro-CT image $\overset{-}{x}$ and micro-CT image $\left\{y_{1}\right\}$, respectively, and minimize their differences, we have:

(7) $$L_{F}\left(G\right)=E_{x,\left\{y_{1},\cdots ,y_{K}\right\}}\left[{\left\|D_{f}\left(\overset{-}{x}\right)-\sum_{k}\frac{D_{f}\left(y_{k}\right)}{K}\right\|}_{1}^{1}\right]$$

The proposed model was trained on a Windows 10 workstation equipped with two Nvidia A6000 GPUs. In the training process, the discriminator G randomly draws two images $c_{x},c_{y}\in S$ and $c_{x}\neq c_{y}$ from different classes of source images (MDCT and micro-CT) and performs mapping training to finally obtain the ability to generate micro-CT-like and MDCT-like images. We used the default hyper-parameters of FUNIT for training but changed the image sizes to fit MDCT and micro-CT images.

#### 3.4.2. Image Pairing Method for Testing

**Figure 4:**
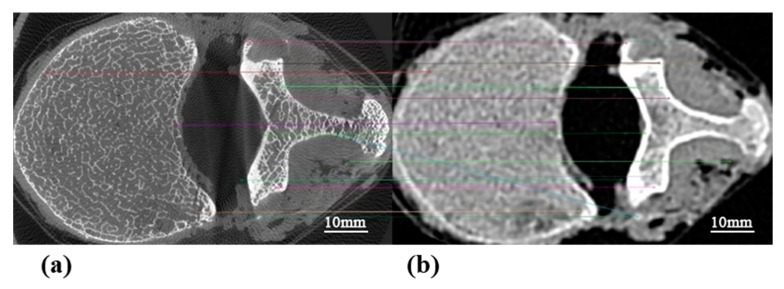
Image matching for micro-CT and MDCT. (**a**) is the micro-CT image, (**b**) is the MDCT image, and the similarity between all micro-CT and MDCT images can be calculated using the average distance of coupling key points. Different colored lines indicate the coupling relationship between key points.

In order to test the performance of the model, a ground-truth image pair set was needed. The scheme for preparing the ground-truth image pair set is as follows:

1. Image matching: The scale invariant feature transform (SIFT) algorithm [78] was used to find coupling key points in MDCT and micro-CT images. We calculated the Euclidean distance between key points and set the mean value to be the distance between MDCT and micro-CT images (Figure 4). Based on this, we compared MDCT and micro-CT images one by one and constructed the matrix of distances between all MDCT and micro-CT images. The best matched image pair could be obtained via the dynamic time warping (DTW) algorithm [79].
2. MDCT image amplification and image pair generation: Due to the different layer spacing between the two scanning methods, MDCT images and micro-CT images of the same specimen are not equal in overall number, and approximately two layers of micro-CT images correspond to one layer of MDCT images. Therefore, the MDCT images of each vertebra needed to be replicated (250×2) according to the matching relationship to obtain one-to-one paired-image pairs of MDCT and micro-CT images, i.e., 500 image pairs were generated for each vertebral specimen. Applying the above method to all 25 vertebrae in the test set, a total of 25×500=12,500 image pairs could be obtained.

### 3.5. Assessment Methods

#### 3.5.1. Similarity Metrics

To evaluate the similarity between two images, this study employed the structural similarity (SSIM) [80] and Fréchet inception distance (FID) [81] metrics. The SSIM is designed to evaluate similarity with respect to structure, where a higher SSIM value signifies greater similarity between images [82]. Conversely, the FID metric focuses on evaluating similarity in terms of details, with a lower FID value indicating a higher degree of similarity between images [83]. The definitions of SSIM and FID can be found in the research [22].

#### 3.5.2. Born Structure Metrics

The trabecular microstructure analysis in micro-CT and micro-CT-like images was conducted by employing the BoneJ plug-in [83] within the Fiji (Version 1.53t) software [83]. Utilizing Fiji, which represents a distribution of ImageJ2 developed by the National Institutes of Health [84,85], both micro-CT and micro-CT-like images of vertebrae were processed as 8 bit stack maps. Then, the gray-level images from micro-CT and micro-CT-like sources were binarized into bone and marrow phases by implementing the IsoData algorithm [86], a global thresholding technique. Following this binarization, metrics were computed, including bone volume fraction (BV/TV), trabecular thickness (Tb.Th) and trabecular spacing (Tb.Sp). BV/TV was derived via simple voxel counting, whereby all the foreground voxels were counted and assumed to represent bone and then compared to the total number of voxels in the image. Tb.Th and Tb.Sp were calculated without model assumptions and measured directly by taking foreground voxels as trabeculae and background voxels as spacing [87].

**Figure 5:**
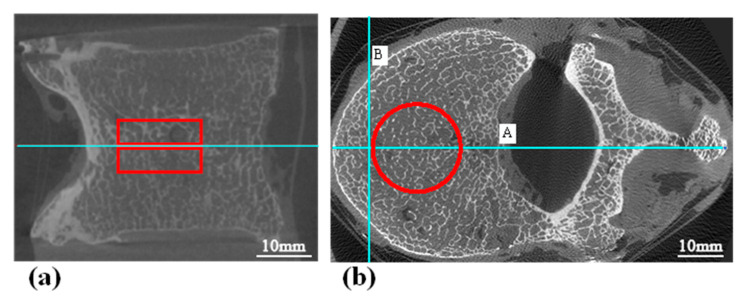
Cylindrical volume of interest (VOI) selection method. (**a**) The sagittal position of the VOI is shown on the vertebral body micro-CT sagittal image via the two areas located 5 mm above and 5 mm below each of the vertebral body sagittal midline. (**b**) The axial position of the VOI is shown on the vertebral body micro-CT axial image. Line A denotes the centerline of the short axis of the vertebral body axial map, line B is perpendicular to line A and the intersection of line A and line B is located 5 mm within the intersection of line A and the anterior edge of the vertebral body. A cylindrical VOI with a diameter of 15 mm was taken with the intersection of line A and line B as the tangent point.

Continuous axial images were required to form a cylindrical volume of interest (VOI) to measure the bone structure metrics. After training the model, all original MDCT images of the 25 vertebrae from the test set were inputted into the model to obtain continuous micro-CT-like images. Subsequently, two cylindrical VOIs (approximately 15 mm in diameter and 5 mm in height) for each vertebra were selected in both the micro-CT and micro-CT-like images. The positioning of the VOI can be found in Figure 5. Identical VOI settings were applied to the MDCT images in order to measure bone structure metrics to serve as a control group.

## 4. Results

### 4.1. Training Results

**Figure 6:**
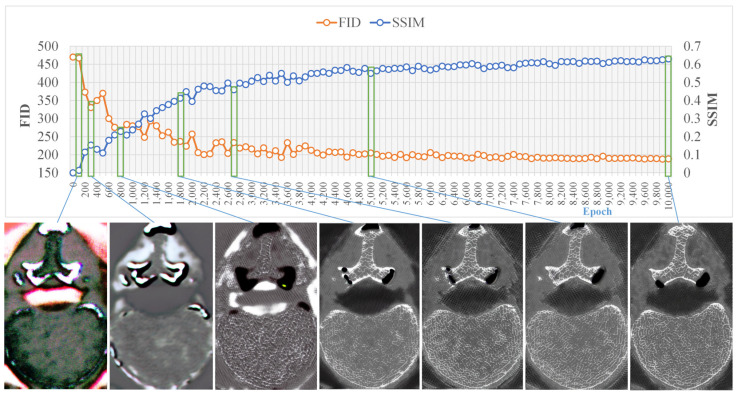
The training effect of the FUNIT model of one slice of vertebra in different epochs. The biaxial line graph at the top of the figure shows the trends of the SSIM value and FID value during training, and the vertebral body images at the bottom are the vertebral micro-CT-like images corresponding to the learning epoch.

Figure 6 shows the process of training by showing metrics of one slice of vertebra in different epochs. After approximately 8000 epochs of learning, the change in the image metrics slowed and stabilized. The figure shows several representative points in the training process, which can be used to observe the learning process of the model for generating micro-CT-like images. The model first learns the contour information of MDCT, starts from the range area, gradually adds the bone cancellous and bone cortical information, and gradually fills in the details of the internal trabecular structure. In the initial stage of the generation process, there are vacant areas, and as the training epoch increases, the vacant areas are gradually reduced and the details of the images are gradually clarified.

**Figure 7:**
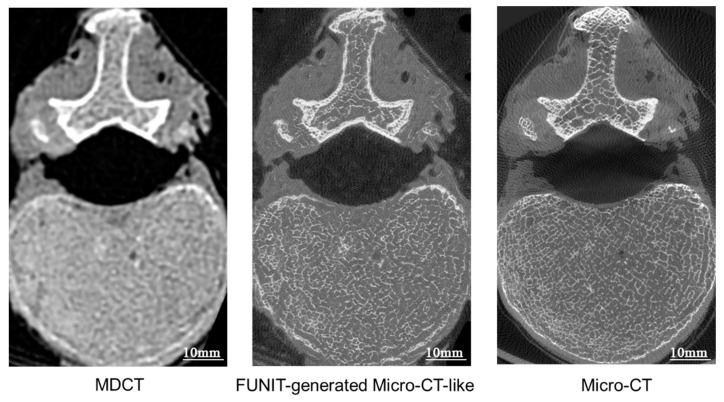
Comparison of FUNIT micro-CT-like image generated from FUNIT, MDCT image and micro-CT image.

**Figure 8:**
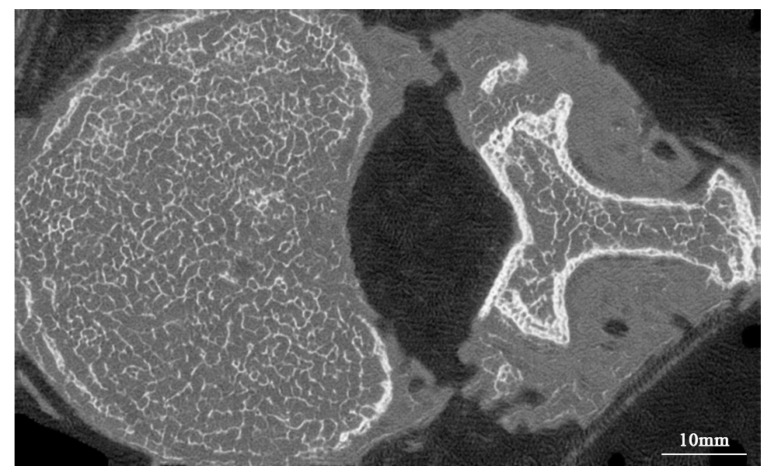
Example of a FUNIT-generated micro-CT-like image.

After training, MDCT images from the test set were input into the unpaired-image-based model to obtain micro-CT-like images. Figure 7 and Figure 8 show examples of micro-CT-like images.

Although the micro-CT-like images have sufficient similarity with gold-standard images, the micro-CT like images have some shortcomings: (1) there is an obvious “double-border phenomenon” in the bone cortex, i.e., the phenomenon of bone cortex delamination; (2) there is a lack of bone cortex on the surface of the vertebral canal; (3) there is a localized trabecular texture in the peripheral soft tissue of the vertebral body; (4) there is a dense area of bone trabeculae in the cancellous bone, and there is an overfilling of bone trabeculae.

### 4.2. Comparison of SSIMs and FIDs for Generated Images

Statistical methods were used to more rigorously determine whether the metrics were significantly different. The normality of all continuous variables was verified using the Kolmogorov–Smirnov test, and chi-squaredness was verified using the Levene test. The Friedman test was used to compare the differences in SSIM and FID values between the images generated using FUNIT, StarGAN and CycleGAN, the original MDCT images and the gold-standard micro-CT images. The Mann–Whitney U test was used to compare the SSIM and FID differences between the FUNIT model and the pix2pixHD model for the micro-CT-like images. The differences in bone structure metrics between the FUNIT micro-CT-like and gold-standard micro-CT images were analyzed using paired *t*-test datasets. Linear regression was used to analyze the correlation between bone structure metrics between the FUNIT micro-CT-like and gold-standard micro-CT images. The Z-test was used to compare differences in correlation coefficients between bone structure metrics between FUNIT micro-CT-like, pix2pixHD micro-CT-like images [22] and micro-CT and MDCT images. Intraclass correlation coefficients (ICCs) were used to analyze the consistency between bone structure metrics between FUNIT micro-CT-like and gold-standard micro-CT images. The above statistical analyses were performed using SPSS 26.0 (SPSS Inc., Chicago, IL, USA) and MedCalcv10.002 (Ostend, Belgium) software, and differences were considered statistically significant if the two-sided *p* value < 0.05. Since the vertebral body consists of cancellous and cortical bone, both of which are of interest for bone strength, we compared the quality of generated images by considering the overall image and local cancellous bone image separately.

#### 4.2.1. Comparing Generated Micro-CT-like Images with MDCT Images

**Figure 9:**
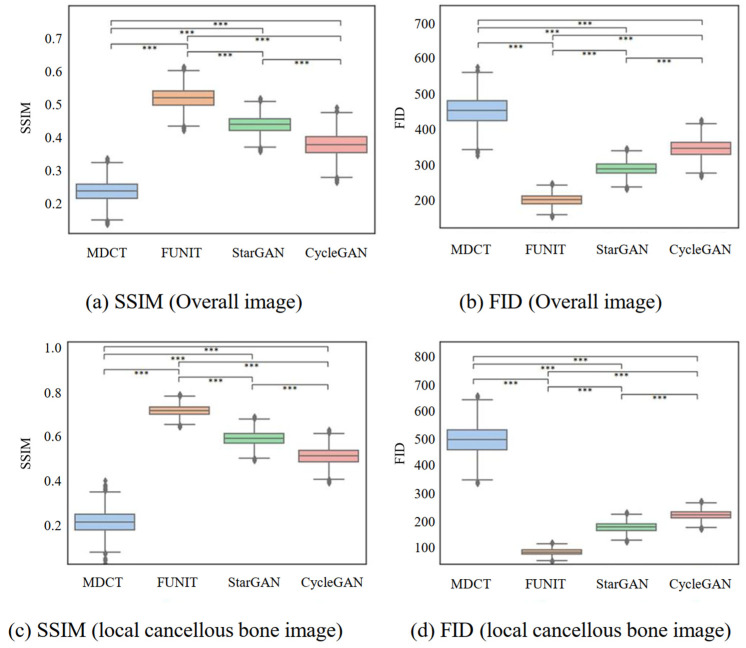
SSIM and FID values of the MDCT and three generated images. The Friedman test was used to test the differences in the metrics between the four groups of images, *** represents p<0.001.

In terms of overall images, using the micro-CT image as the gold standard, the mean values of SSIM between gold-standard images and the micro-CT-like images generated by using three unpaired-image-based models (i.e., FUNIT, StarGAN and CylceGAN) were greater than the SSIM values between the gold-standard and MDCT images, and the differences were statistically significant (p<0.001). Similarly, using micro-CT as the gold standard, the FID values of the generated images were all smaller than the FID values of MDCT. The differences were statistically significant (p<0.001), and these results are shown in Table 1 and Figure 9. Based on these, we found that the micro-CT-like images generated using the three unpaired-image-based models were more similar to the gold-standard images than the original MDCT images in terms of macro-structure and detailed micro-structure. Among the three unpaired-image-based models, the metrics (both SSIM and FID) of the micro-CT-like images generated using FUNIT were better than those of the other two comparison models, and the differences were statistically significant (p<0.001).

In terms of localized cancellous bone images, the mean values of SSIM and FID of generated micro-CT like images generated by the three unpaired-image-based models improved compared with the values of the overall image. Additionally, FUNIT performed better than the other two methods in SSIM and FID, with statistically significant differences (p<0.001).

#### 4.2.2. Comparison of Micro-CT-like Images Generated Using Unpaired-Image-Based FUNIT Model and Paired-Image-Based pix2pixHD Model

**Figure 10:**
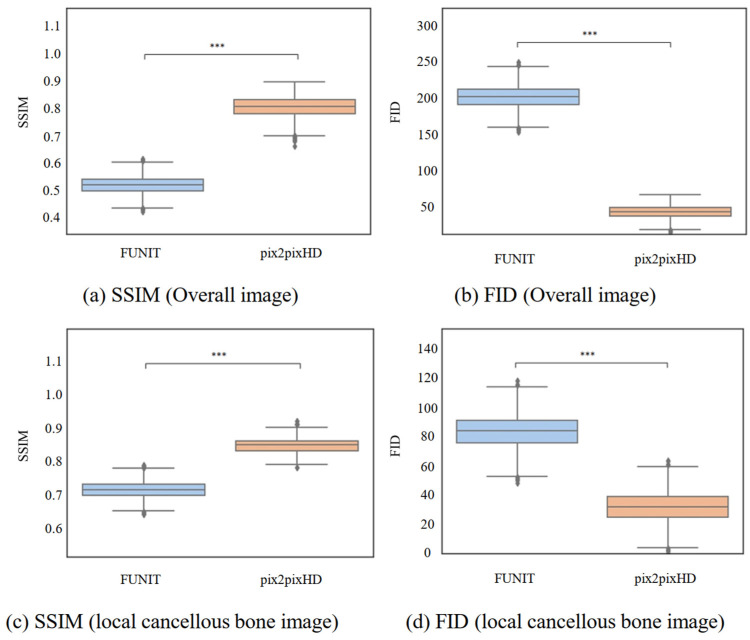
SSIM and FID values of the generated images of paired-image-based pix2pixHD and unpaired-image-based FUNIT. The Mann–Whitney U test was used to test the differences in metrics between the images. *** represents p<0.001.

In terms of both the overall image and the local cancellous bone image, the SSIM and FID values of the FUNIT-generated micro-CT-like images were better than the correlation values of the pix2pixHD-generated micro-CT-like images, and the differences were statistically significant (p<0.001). These results are shown in Table 2 and Figure 10.

### 4.3. Correlation and Consistency of Bone Structure Metrics between Generated Micro-CT-like and Gold-Standard Micro-CT Images

#### 4.3.1. Correlation of Bone Structure between FUNIT-Generated Micro-CT-like and Gold-Standard Micro-CT Images

The bone structure metrics of FUNIT-generated micro-CT-like and gold-standard micro-CT images with their correlations are shown in Table 3. The correlation values of BV/TV and Tb.Th of FUNIT-generated micro-CT-like images were smaller than those of the gold standard, while the Tb.Sp was larger than that of the gold standard, and the difference was statistically significant (p<0.001). Linear regression equations for bone structure metrics of FUNIT-generated micro-CT-like and micro-CT images were: BV/TV: y=0.935x−0.025; Th.Th: y=1.078x−0.076 and Tb.Sp: y=1.029x+0.182, with $R_{\left(FUNIT\right)}^{2}$, and the F values are shown in Table 3. The BV/TV, Tb.Th and Tb.Sp values of FUNIT-generated micro-CT-like images were highly correlated with those of the gold standard, and the correlation was significant (p<0.001).

#### 4.3.2. Consistency between Bone Structure Metrics of FUNIT Micro-CT-like and Gold-Standard Micro-CT Images

The ICC values of the bone structure metrics of FUNIT-generated micro-CT-like and gold-standard micro-CT images are shown in Table 4. The FUNIT-generated micro-CT-like bone structure metrics are highly consistent with those of the gold standard.

### 4.4. Discussion

#### 4.4.1. Characterization of the Proposed Method

From both the overall image and the local cancellous bone image, the SSIM values of the micro-CT-like images generated using the three unpaired-image-based methods were greater than those of MDCT, and the FID values were smaller than those of MDCT (p<0.001). The micro-CT-like images generated using the unpaired-image-based methods had more obvious improvements in structure and details than the original MDCT images, and the generated micro-CT-like images were more similar to the gold-standard images. Comparing the results of three unpaired-image-based models, we found that the FUNIT method had larger SSIM values and smaller FID values than the other two unpaired-image-based models (p<0.001), indicating that the FUNIT method had the best model performance in the image mapping process among the three groups of models.

FUNIT focuses on generating structured images and uses a more systematic generator design, which consists of three main parts: a content encoder and a class encoder and decoder. The content encoder extracts information from MDCT that is not related to detailed texture but highly relevant to the location and regional structure, such as the structure of each region in cancellous bone and the macro layout of bone trabeculae. Then, a content feature code is generated after extraction. The class encoder learns location-independent bone trabeculae detail information from micro-CT, including texture, alignment, etc. The class specific features are generated after extraction [23]. The model simultaneously learns the mapping relationship between MDCT and micro-CT and finally fuses the class features with the content features on the decoder to form micro-CT-like images. Thus, hidden information such as bone material and bone marrow distribution in MDCT is extracted, and bone trabeculae texture is attached to form micro-CT-like images. By the judgment of the discriminator, the formed micro-CT-like image will have the characteristics of the bone trabecular structure in micro-CT. For this reason, FUNIT can perform better in the environment studied in this paper and generate micro-CT-like images that exceed those of other unpaired-image-based methods in quality.

**Figure 11:**
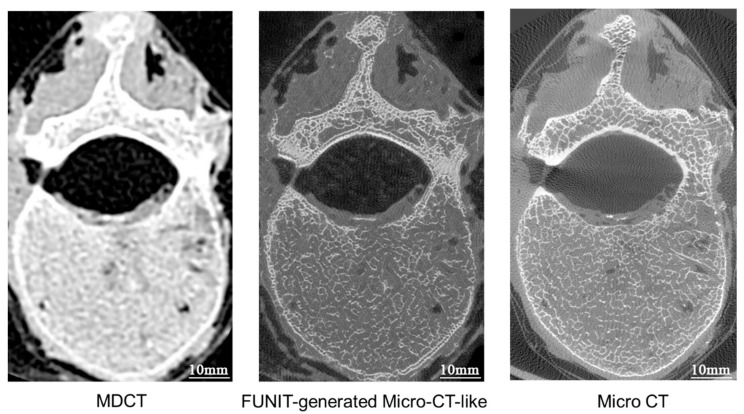
Comparison of FUNIT-generated micro-CT-like, MDCT and micro-CT images.

**Figure 12:**
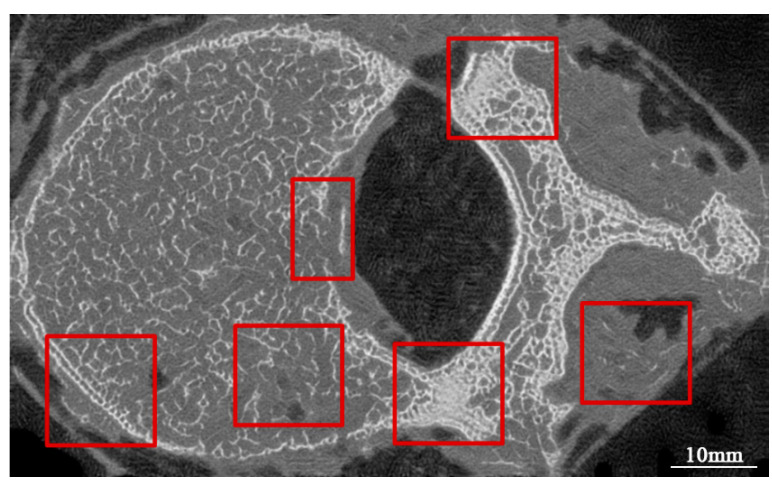
Examples of defects in micro-CT-like images generated via FUNIT. The red boxes are the areas where anomalies exist.

Although the FUNIT model used can generate micro-CT-like images that are more similar to the gold standard than the other two methods, the generated images still have deficiencies. From the results, the SSIM value of the image of the cancellous bone portion of FUNIT-generated micro-CT-like was improved compared to the overall image, indicating that the cancellous bone region was more similar to the gold standard, while there were some problems in the outer contour of the vertebral body, i.e., the bone cortex. Figure 11 shows an example of FUNIT-generated micro-CT-like images, and the problems with the images are shown specifically in the red box in Figure 12. First, there is a clear “double-border phenomenon” in the bone cortex, where the originally compact bone cortex is filled with two or more layers of thin linear bone cortex. The possible reason for this phenomenon is that the model focuses on cancellous bone features when generating the images, and the whole image is filled with the structural pattern of bone trabeculae, so the bone cortex on the MDCT image is replaced by multiple near-parallel bone trabeculae textures.

Additionally, there is a problem of loss of bone cortex in specific areas, especially in the vertebral canal surface where the bone cortex is prone to defects and disruption of continuity, which in turn leads to a situation where the boundary between the bone tissue and the surrounding soft tissue is unclear.

Furthermore, short trabecular texture-like shadows of bone trabeculae appear within the peripheral soft tissues of the vertebral body. This is due to texture within the soft tissues being mistaken for bone trabeculae in MDCT: soft tissues with discrepancies in CT values may be misidentified as bone tissue and then filled. However, this phenomenon is not widespread and does not have an impact on bone structure studies.

Finally, in the case of vertebral cancellous bone, if there are relatively dense areas in the cancellous bone, FUNIT will overfill the relatively dense areas to a certain extent during the mapping process, as shown by the local thickening of the trabeculae. In contrast, the relatively sparse areas are underfilled, which is reflected by the local absence and thinning of trabeculae.

Although there are some issues in the micro-CT-like images generated via FUNIT, none of them are distributed in core regions of cancellous bone. This is the reason why the localized SSIM and FID values were better than the overall SSIM and FID values. Since cancellous bone is important for the diagnosis of osteoporosis, it can be assumed that the studied FUNIT method meets the requirements of bone structure analysis.

The BV/TV and Tb.Th of FUNIT-generated micro-CT-like images were smaller than those of the gold standard, and the differences were statistically significant (p<0.001). The Tb.Sp of FUNIT-generated micro-CT-like images was greater than that of the gold standard, and the difference was statistically significant (p<0.001). All measured bone structural metrics were moderately correlated with the gold standard (BV/TV: $R_{\left(FUNIT\right)}^{2}=0.667$, Th.Th: $R_{\left(FUNIT\right)}^{2}=0.613$, Tb.Sp: $R_{\left(FUNIT\right)}^{2}=0.603$), the correlation was higher than that of MDCT (BV/TV: $R_{\left(MDCT\right)}^{2}=0.367$, Th.Th: $R_{\left(MDCT\right)}^{2}=0.275$, Tb.Sp: $R_{\left(MDCT\right)}^{2}=0.283$) and the differences were statistically significant. The ICC results showed that acceptable consistency existed between the generated images and the gold standard. However, the smaller BV/TV and Tb.Th and larger Tb.Sp imply that the trabeculae are broken, missing, or unfilled during the mapping process, resulting in wider spacing and a relative decrease in bone volume fraction. This situation may occur because FUNIT is obtained by finding structures in MDCT and later adding details similar to those in micro-CT images to obtain micro-CT-like images. If the structure in MDCT is not very obvious, details are easily missed and the results of its generated images will be biased toward conservatism. On the other hand, the unpaired-image-based method learns the structure in MDCT corresponding to the texture feature in the micro-CT image, and this feature is not learned one-to-one, meaning that unreasonable bone trabeculae orientation, etc., may occur when filling the details. This result may lead to a reduction in predicted bone strength compared to actual bone strength when FUNIT-generated micro-CT-like images are eventually used to predict bone strength, which in turn may lead to an increased false-positive rate in fracture risk prediction. The further optimization of model parameters and increased sample diversity are needed in subsequent studies to remedy this deficiency.

#### 4.4.2. Paired-Image-Based pix2pixHD Model versus Unpaired-Image-Based FUNIT Model

**Figure 13:**
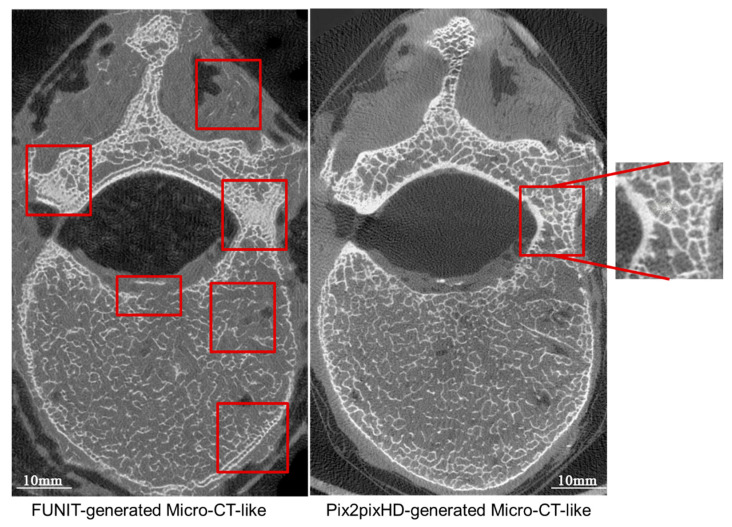
Comparison of micro-CT-like images generated using the paired-image-based pix2pixHD method with those generated using the unpaired-image-based FUNIT method. The red boxes are the areas where anomalies exist.

By comparing the SSIM with the FID index, as well as the direct sample shown in Figure 13, we found that the pix2pixHD-micro-CT-like images were more similar to the gold standard than the FUNIT-micro-CT-like images. FUNIT generates less of the bone cortex and is prone to problems such as the “double-border phenomenon” on the bone cortex, missing bone cortex and trabecular texture in the soft tissue. In contrast, the bone cortex of pix2pixHD-generated images is more similar to that of the micro-CT gold standard, with a tighter and more continuous bone cortex and a clear boundary with the soft tissues. As analyzed, this is related to the training mechanism of FUNIT and pix2pixHD, which adopts a “class-to-class” learning model and has a certain tendency to “imagine” in the filling process, i.e., it uses the local information of MDCT for generation. In contrast, the pix2pixHD method adopts an “image-to-image” learning mode, and its “imagination” capability is more convergent; consequently, the mapping results are more realistic, which is one of the advantages of paired-image training. However, pix2pixHD-generated micro-CT-like images also have the problem of overfilling and noise formation in dense and complex bone areas such as attachments. Although there are still some shortcomings in the texture details of both methods, such as reduced local trabecular definition and less natural alignment, which make the measured bone structure metrics not fully consistent with those of the gold standard, there is sufficient correlation between the bone structure metrics and those of the gold standard.

Unpaired-image-based learning does not require paired images due to its learning mechanism, and it has a greater ability to generalize. The model can find the structural features embedded in MDCT images and find their mapping relationships with micro-CT images to make certain associations and add detailed textures. This property allows the model to transform images to a limited degree even when it encounters MDCT input data of a vertebra type that has not appeared before, making the trained model somewhat robust.

## 5. Conclusions

As the population ages and life expectancy continues to increase, the incidence of fragility fractures has increased significantly. Therefore, the early identification of fragility fracture risk is critical. In addition, as the age of the population treated with spinal instrumentation increases, clinicians need to pay more attention to bone strength profiles to develop individualized surgical plans and reduce the probability of postoperative complications. BMD cannot fully explain changes in bone strength alone, so it is extremely important to analyze a diversity of bone structure metrics. The primary focus of this study is to investigate the possibility of measuring vertebrae bone structure metrics using MDCT images, of which the core task is establishing a mapping relationship between vertebral MDCT images and micro-CT images using deep learning methods to generate micro-CT-like images based on MDCT images.

From the perspective of computer image science, mapping two images with vastly different resolutions, such as MDCT and micro-CT images, remains an open research challenge. The emergence of CGANs and their derived models has made this feasible [17]. In this study, the above image mapping task was achieved by finding nonlinear feature associations between vertebral MDCT and micro-CT images through the unpaired-image-based FUNIT method.

The bone structure metrics measured using micro-CT-like images are highly correlated with those obtained from the gold standard of micro-CT images. The used method can fully utilize the potential of MDCT images and provides a technical methodological possibility to realize in vivo vertebral bone structure measurement. In terms of image translation, this paper discusses the presence of some phenomena (e.g., the double-border phenomenon), but it mainly focuses on the qualitative discussion. Quantitative description methods of these phenomena should be explored in depth in the future. In terms of model training, although it is currently in the preliminary exploratory stage using a small sample of in vitro vertebral specimens, the deep learning model can be further optimized, and its generalization capability can be improved in the future through measures such as expanding the sample size, increasing sample diversity, and simulating in vivo environments. More detailed and systemic clinical evaluations should be conducted in the future.

## Figures and Tables

**Table 1:** SSIM and FID values of the four sets of images and the gold-standard micro-CT images.

Scale	Metrics	MDCT	FUNIT	StarGAN	CycleGAN	*p*-Value †
Overall image	SSIM	0.238 ± 0.031	0.519 ± 0.030	0.437 ± 0.025	0.377 ± 0.035	<0.001 ***
	FID	453.425 ± 39.081	201.737 ± 15.031	289.503 ± 18.037	347.311 ± 25.051	<0.001 ***
Localized cancellous bone images	SSIM	0.213 ± 0.052	0.714 ± 0.023	0.589 ± 0.031	0.508 ± 0.037	<0.001 ***
	FID	495.024 ± 54.435	83.696 ± 11.022	175.531 ± 17.035	219.559 ± 16.033	<0.001 ***

Note: † The Friedman test was used to test the differences in metrics among the four sets of images; *** indicates the corresponding image quality evaluation indicators compared between groups have p<0.001.

**Table 2:** Comparison of the micro-CT-like images generated using the FUNIT model and pix2pixHD model.

Scale	Metrics	FUNIT	pix2pixHD [22]	*p*-Value †
Overall image	SSIM	0.519 ± 0.030	0.804 ± 0.037	<0.001 ***
	FID	201.737 ± 15.031	43.598 ± 9.108	<0.001 ***
Localized cancellous bone images	SSIM	0.714 ± 0.023	0.849 ± 0.021	<0.001 ***
	FID	83.696 ± 11.022	31.724 ± 10.021	<0.001 ***

Note: † The Mann–Whitney U test was used to verify the differences in metrics between micro-CT-like images generated using FUINT and pix2pixHD. *** indicates p<0.001.

**Table 3:** Bone structure metric values and correlation between FUNIT-generated micro-CT-like and micro-CT images.

N=50	FUNIT Micro-CT-like	Micro-CT	*p*-Value †	$R^{2}$	F-Value	*p*-Value ‡
BV/TV (%)	0.143 ± 0.018	0.180 ± 0.016	<0.001 ***	0.667	96.102	<0.001 ***
Tb.Th (mm)	0.158 ± 0.021	0.218 ± 0.015	<0.001 ***	0.613	78.69	<0.001 ***
Tb.Sp (mm)	1.144 ± 0.166	0.934 ± 0.126	<0.001 ***	0.603	75.573	<0.001 ***

Note: † Paired *t*-test was used to compare the difference between the two groups of bone structure metrics, *** represents p<0.001. ‡ Linear regression was used to analyze the correlation between the two groups of bone structure metrics, *** represents p<0.001.

**Table 4:** ICC values of bone structure metrics of FUNIT-generated micro-CT-like and gold-standard micro-CT images.

	Bone Structure Metrics	ICC	95% CI	*p*-Value
micro-CT-like (FUNIT). vs. micro-CT	BV/TV	0.809	0.887~0.686	<0.001
	Tb.Th	0.752	0.852~0.601	<0.001
	Tb.Sp	0.753	0.852~0.603	<0.001

## Data Availability

Data sharing not applicable.
